# Systematizing Audit in Algorithmic Recruitment

**DOI:** 10.3390/jintelligence9030046

**Published:** 2021-09-17

**Authors:** Emre Kazim, Adriano Soares Koshiyama, Airlie Hilliard, Roseline Polle

**Affiliations:** 1 Department of Computer Science, University College London, Gower St, London WC1E 6EA, UK; a.koshiyama@cs.ucl.ac.uk (A.S.K.); roseline.polle.19@ucl.ac.uk (R.P.); 2 Institute of Management Studies, Goldsmiths, University of London, New Cross, London SE14 6NW, UK; ahill015@gold.ac.uk

**Keywords:** transparency, accountability, governance, compliance, robustness, explainability, privacy, bias, fairness, recruitment

## Abstract

Business psychologists study and assess relevant individual differences, such as intelligence and personality, in the context of work. Such studies have informed the development of artificial intelligence systems (AI) designed to measure individual differences. This has been capitalized on by companies who have developed AI-driven recruitment solutions that include aggregation of appropriate candidates (*Hiretual*), interviewing through a chatbot (*Paradox*), video interview assessment (*MyInterview*), and CV-analysis (*Textio*), as well as estimation of psychometric characteristics through image-(*Traitify*) and game-based assessments (*HireVue*) and video interviews *(Cammio*). However, driven by concern that such high-impact technology must be used responsibly due to the potential for unfair hiring to result from the algorithms used by these tools, there is an active effort towards proving mechanisms of governance for such automation. In this article, we apply a systematic algorithm audit framework in the context of the ethically critical industry of algorithmic recruitment systems, exploring how audit assessments on AI-driven systems can be used to assure that such systems are being responsibly deployed in a fair and well-governed manner. We outline sources of risk for the use of algorithmic hiring tools, suggest the most appropriate opportunities for audits to take place, recommend ways to measure bias in algorithms, and discuss the transparency of algorithms.

## 1. Introduction

Algorithmic systems that draw on business psychology literature studying workforce intelligence have the potential to revolutionize hiring through AI-driven recruitment tools (Hadjimichael and Tsoukas 2019). Broadly, the workplace can be thought of as a defined space of interactions, with agents (staff and managers) and inputs/outputs (business performance metrics and other relevant performance indicators) that can be assessed together (Hadjimichael and Tsoukas 2019). Study of this space has both explicit and implicit dimensions, where the former refers to occurrences and processes that are explicated and objectified through interviewing, documentation, and other articulated management schemes. The latter, implicit, is characterized by occurrences and processes that are present but not clear or articulated (‘unconscious’), and this ‘tacit knowledge’ can be assessed in AI-driven recruitment^1^ (Bender and Fish 2000; Davenport 2018; Davenport and Kalakota 2019; Woolley et al. 2015; Wright and Atkinson 2019). 

Notwithstanding the opportunities that this technology can offer, there is increasing concern about the ethics of using AI in recruitment, particularly due to the uproar caused by Amazon’s AI recruitment tool, which was biased against female applicants, resulting in Amazon retiring the technology (Dastin 2018). In response to these concerns, there is a movement toward addressing how AI can be used in a responsible and fair way, with this movement being characterized by three broad phases (Kazim and Koshiyama 2020b):

- Principles: A large number of academic groups, governmental bodies, private industry and non-governmental organizations have published their own principles for the ethical use of AI (European Commission 2021; Kazim et al. 2021; Piano 2020);
- Processes: The second phase, typified by an ethical-by-design approach, was an engineering focused problem-solving exercise. An important dimension of this phase was the recognition that factors such as governance, social impact, legal compliance, and engineering standards should be considered during the processes involved in the creation and introduction of AI technologies (Leslie 2019);
- Assurance and audit: We believe the third phase (and current one) is concerned with the need to standardize the use of AI, where such standardization can be in the form of legal compliance, meeting industry best practice, or some sector-specific regulation (Kazim et al. 2021).

We believe that auditing will be critical to assuring the integrity of algorithmic systems, particularly those used in recruitment, with so-called ‘AI audits’ likely to become mandatory. These audits would assess the technologies according to trusted standards and frameworks concerned with practices including impact assessments, data protection, governance, and reporting. To address this, there has been a recently proposed systematic approach to the auditing of machine learning, artificial intelligence and associated algorithmic systems (see Koshiyama et al. 2021). In the sections below, after introducing AI-driven recruitment and the innovation that is occurring in this field, we expand upon how this auditing framework (Koshiyama et al. 2021) can be applied to algorithmic recruitment systems that have been informed by the work of business psychologists. 

Before applying the said framework in the context of AI-driven recruitment systems, we note that the principal reason for the use of this framework is that the other approaches (such as those that fell under the ‘principles’ (European Commission 2021; Kazim et al. 2021; Piano 2020) and ‘processes’ (Leslie 2019) phases (discussed above)) fail to take into account that AI governance must span both the non-technical (articulation of principles, allocation of responsibility, training, documentation, etc.) and the technical (accuracy, security, bias, etc.). Furthermore, the approach we take is holistic, insofar as the framework we apply spans levels of access (depth of audit), the point at which an audit should take place (locating when an audit can take place and how that point in a process impacts the nature of the audit that can be performed) and critically covers technical verticals (bias, privacy, etc.) as well as a route to what would be appropriate documentation. Comparatively, both Arslan (2000) and Raji et al. (2020) discuss AI audits in terms of integration into the business process, which we recognize is a critical component of making audits useful in practice. However, the work does not touch on the specific technical verticals that need to be assessed (explainability, robustness, etc.). Similarly, the approaches to audits that are found in Mokander and Floridi (2021), Munoko et al. (2020) and Umbrello and van de Poel (2021) lack engagement with AI engineering practice and the literature itself and are thus incomplete, yet at this time form the technical perspective.

## 2. Auditing AI-Driven Recruitment Systems

### 2.1. Algorithmic Recruitment

Recruitment is a clear example of workplace intelligence that has a high impact, both in terms of ethics due to concerns about fair hiring, and in terms of business due to wanting the best hires. Since the arrival of the digital age, the internet has become an invaluable resource for both job hunters and recruiters. Indeed, online job boards advertising job listings first emerged in the 1990s and still continue to evolve today (Real-Time Talent 2016), with the expanding job market promoting the development of more targeted recruitment tools (Real-Time Talent 2016). An example of this is machine learning techniques, which automatically screen resumes to select qualified applicants for an advertised position by using information about their professional competence and human skills to predict whether they satisfy the basic characteristics and skills required for the role. These models are based on training sets that use features and outcomes extracted from past data to construct a decision rule that is designed to be well-generalizable to future applicants. If a candidate is not considered to have met the minimum requirements for the position, they are automatically rejected and are not invited to progress to the next step in the recruitment process.

Companies have started to take advantage of similar machine learning algorithms to offer other AI-driven recruitment solutions that can extract relevant information about candidates.^2^ For example, *Hiretual* offers an AI-based candidate search tool that allows their customers to compile a database of suitable contenders based on a search across multiple applicant platforms. *Textio* also makes use of AI with their augmented writing platform that gives hiring managers guidance on the most effective language to use in job adverts, while *Paradox* uses a virtual assistant to conduct chat-based interviews to screen applicants, and *MyInterview* examines the professionalism and authenticity displayed by candidates during video interviews.

Other AI-based recruitment systems focus on the psychometric characteristics of candidates, with *Traitify* offering image-based measures of personality and *HireVue* using game-based versions of traditional psychometric assessments to estimate attributes such as personality, intelligence and emotional intelligence. Additionally, companies such as *Cammio* offer AI-based tools to analyze video interviews and predict psychological traits such as personality, with these psychometric traits being able to predict how well applicants will perform in a future role (Schmidt and Hunter 2016). The models used in these recruitment solutions are typically informed by the work of business psychologists who often work closely with the developers of these tools to create algorithms that can predict how a hiring manager would evaluate a candidate, reducing the need for psychologists or trained recruiters to be involved in assessing applicants.

Such technologies can bring benefits to both hiring managers who no longer have to manually screen a large number of resumes through an inefficient and subjective process, and candidates who can receive an instant response to their application. Algorithmic hiring, therefore, has significant value in recruitment through significantly reducing the time and cost of hiring. Through reasonable modeling, the automatic hiring tools can make more impartial and rational decisions. At the same time, they can respond to new data input and modify the decision rules in a timely manner in order to improve the quality of hiring. However, as these hiring tools are typically only used to screen candidates, with the ultimate hiring decisions being left to the hiring manager, they do not completely eliminate the need for human involvement (Rieke et al. 2018). As recruitment decisions are crucial for both the employer and the applicant, the criterion for decision making should be measured deliberately.

### 2.2. Discrimination in Algorithmic Hiring

Crucially, concerns about the potential for discrimination resulting from AI-driven hiring tools echo concerns about the lack of impartiality in human hiring decisions; remarkable discrimination against female and black workers was identified on the Freelancer platforms TaskRabbit and Fiverr (Hannák et al. 2017). While advanced hiring platforms consciously remove protected information such as gender and address from resumes to reduce biases (Real-Time Talent 2016), this is insufficient because the protected attributes could be encoded in the insensitive details presented in the resume (Pedreshi et al. 2008). 

This has already been seen to cause issues such as in the case of Amazon’s retired recruitment tool, which penalized resumes that included the word “women’s” (Dastin 2018). Before expanding further, it is important to note that bias in AI-driven systems can occur for a number of reasons, including bias in the training data, model parameters and the process of development (team, etc.)—the most well-known source is data bias, where a ‘junk in—junk out’ principle occurs (Mehrabi et al. 2021).

Just as with the traditional recruitment process, discrimination in algorithmic hiring can take one of two forms: direct discrimination and indirect discrimination. Direct discrimination occurs when protected attributes such as gender and race are explicitly taken as features in the decision-making process, resulting in discrimination against those belonging to specific groups. Indirect discrimination, on the other hand, is more common and, as in the case of Amazon’s model, results from the extraction of protected information encoded in seemingly fair data such as education and housing (Ajunwa et al. 2016). Thus, despite the developers of some hiring tools deliberately removing sensitive information from applications before they are processed, the reality is that the discrimination still exists. In addition, those who belong to a minority group may be overlooked when developers assess the performance of the model, meaning that a model that appears to be highly accurate when tested on majority groups may produce high error rates in these populations. Consequently, unintentional discrimination could occur if we simply choose the best model according to the overall accuracy without examining its performance for specific groups.

Algorithmic hiring is not just a transient fad (Ajunwa et al. 2016); it is valued and applied by many large companies and is likely to continue to evolve and become a mainstream recruitment tool. The Covid-19 pandemic has also accelerated this automation, resulting in the rapid development and deployment of AI in many sectors (See Cedefop (2020)). It is, therefore, important to address the potential social and ethical issues that can arise from the use of these tools. This is the aim of the U.S. Equal Employment Opportunity Commission (EEOC), a federal agency that enforces laws relating to workplace discrimination that organizations must comply with. These laws require organizations applying algorithms in their recruitment process to use them responsibly, thereby reducing the potential for negative public opinion or the incorrect dismissal of qualified applicants, which may arise from biased algorithms. The EEOC also suggests that applicants should have the right to know why their application was rejected to protect them against rejection due to discriminatory factors such as age, gender, or ethnicity, and allow them to take action if this does occur. However, this movement towards unbiased hiring is restricted, as discriminatory hiring is currently difficult to prove due to a lack of transparency.

## 3. Assessing AI-Driven Recruitment Systems through Audit

In order to safeguard minority candidates and ensure trustworthiness of algorithms, mandatory auditing of AI-driven recruitment services has been called for (European Commission 2021). Indeed, as of April 2021, New York City policymakers were debating (Int 1894–2020) whether there should be mandatory ‘bias audits’ for such systems, and the EU has published proposed legislation that explicitly names recruitment as a high-risk use of AI requiring a risk-management system (European Commission 2021; Kazim and Koshiyama 2021). These proposals have informed the audit framework we provide below and should be read as expanding current professional body guidelines and labor laws (c.f. US EEOC).

Crucially, audits and assessments should follow a predefined standard or methodology to ensure that there is consistent classification of acceptability (accuracy thresholds, bias metrics, transparency mechanisms, etc.). We define algorithm auditing as ‘the research and practice of assessing, mitigating, and assuring an algorithm’s legality, ethics and safety’. This algorithm auditing work draws from other areas, such as ethics, law, software security and certification (Hagendorff 2020; Jobin et al. 2019; Kazim et al. 2021; Pasquale 2019; Robertson et al. 2018; Rushby 1988; Ryan 1982; Shneiderman 2016; Voas and Miller 2006). As will become clear in the following, we concentrate on mechanisms of assessing the performance of a system through the perspective of an engineering-focused technical audit. A comprehensive audit, however, should encompass the entire pipeline of the system’s life cycle, addressing areas such as the reporting and justification of the business case, assessment of the developing team, and test datasets. In other words, we recognize that technical assessments will form a critical dimension of a wider auditing process. As such, in this section we draw upon the AI Audit framework proposed by Koshiyama et al. (2021) and apply this to the ethically critical domain of recruitment.

### 3.1. Risk

With respect to AI-driven recruitment tools, there are a number of strategic risks that a developer or procurer of the service may face.

- Compliance risk: A system should not contravene the laws within which it is operating. There are several laws that may be applicable, including labor laws, antidiscrimination legislation (Civil Rights Act of 1964), and right to redress (EU GDPR). Failure to comply with these laws risks litigation;
- Reputational: As evidenced by the fallout and public concern following cases of high-profile harm (cf. bias in Amazon’s AI recruitment), companies that are seen to have unethical recruitment practices are liable for reputational damage;
- Financial: Financial loss can be incurred as a result of fines or lawsuits initiated by customers and regulators, loss of commercial earnings through customer loss, and costs associated with poor recruitment strategies such as hiring the wrong candidate, missing out on top performers, and poor staffing strategies;
- Governance: System developers and deployers should always be in control of the systems and have the ability to monitor and report on these systems. The use of such technologies increases risk of loss of control; hence, good accountability measures, such as reporting and documentation, are required.

All of the above, which is a non-exhaustive list, require employing adequate governance structures that can manage these risks and thereby mitigate the potential harm associated with the use of AI-driven recruitment tools. More speculatively, we argue that where the last decade had a focus on ‘data privacy’, the current decade will focus on ‘algorithm conduct’ (Koshiyama et al. 2021).

### 3.2. Auditing Stages

Comprehensive audits assess various aspects of the model at different stages. With respect to recruitment systems, the relevant opportunities for audits are as follows (Koshiyama et al. 2021):

- Data: Input data are analyzed to ensure that they do not include any identifiable or protected characteristics (or proxy for those). Output data are used to compute performance and fairness metrics for different disadvantaged groups, as well as the robustness of the outputs under different perturbations (e.g., noise, removing features);
- Model: The model choice, chosen parameters and objective function influence the explainability and robustness of the process. The auditor will also look for signs of overfitting that could hamper the generalization capabilities of the model and limit how applicable the model is outside of the population it was trained on;
- Development: The design, building process, training routine of the algorithm and associated documentation are audited using information relating to the anonymization process, the reasons why certain features are used, and whether there is any adverse impact analysis to identify whether the model results in group differences and at what stage this analysis takes place.

### 3.3. Verticals

Verticals relate to the performance of a system in terms of bias, transparency, safety, and privacy. These verticals are derived from a maturing of the engineering literature referred to in terms of ‘AI ethics’ and ‘trustworthy AI’ (Kazim and Koshiyama 2020a; Hagendorff 2020; Bartneck et al. 2021; Dignum 2018). In the context of recruitment, these verticals can be understood in the following way:

- Bias: Ensuring systems act fairly across protected characteristics. This is perhaps the most acute vertical of concern, as recruitment is a sector that impacts an individual’s life prospects. Indeed, that a system is fair is not only an ethical imperative but also a legal one. There are a number of bias metrics that can be used to assess how the models impact unprivileged or minority groups (see IBM Research 2021; Cohen 1988; Morris and Lobsenz 2000):
  ○ Disparate Impact: Ratio of the selection rates of the unprivileged group to the privileged group, where selection rates refer to the proportion of candidates belonging to a specific group that are hired;
  ○ Statistical Parity: This compares the selection rates of the unprivileged and privileged groups to determine whether a prediction is independent of a protected characteristic. If statistical parity occurs, the selection rates of the privileged and unprivileged group would be the same;
  ○ Cohen’s D: Effect size used to indicate the standardized difference in the selection rates of the unprivileged and privileged groups. Similar to Statistical Parity but divided by the pooled standard deviation of the selection rate of the two groups;
  ○ 2-SD Rule: If the standard deviation (SD) difference between the expected and observed selection rates for a specific group is greater than 2 SDs, this would be considered as unfair;
  ○ Equal Opportunity Difference: The difference between the true positive rates of the unprivileged and privileged groups. The true positive rate measures the proportion of candidates that are correctly allocated to an outcome by the model, as determined by checking against an alternative model or process such as manual applicant screening;
  ○ Average Odds Difference: Average difference between the false positive rate and true positive rate between unprivileged and privileged groups. The false positive rate measures the proportion of candidates that are supposed to receive an unfavorable outcome, as determined by an alternative measure, but are instead given a favorable outcome by the model.
- Transparency: This vertical is bidimensional, encompassing transparency in the governance and decision-making procedures, and system explainability. With respect to the former, relevant areas for transparency include documentation and standardization of assessments, while the latter is concerned with the extent to which the algorithmic system can be explained. In engineering literature, the degree of transparency is referred to as a black- or white-box, where black-box models lack transparency, with nothing being able to be explained. Indeed, this relates to the broad area known as explainable AI (often referred to as XAI) (Adadi and Berrada 2018; Arrieta et al. 2020). In terms of the recruitment context, good practice entails being able to provide a reason for why a candidate was not hired and, in doing so, enabling the candidate to understand why and potentially improve in that dimension. Furthermore, users of the system can be aware of why the system has recommended in the manner in which it has, and thus whether the reasoning was legitimate (and fair);
- Safety (also referred to as Robustness): A safe and robust model is one that is accurate when applied in different contexts or to different datasets. While a motivating factor in the use of such technologies is increased efficiency, it is critical that the models can be applied fairly across all candidates to avoid unfair decisions and filtering out the best candidates. This is critical given that for a system to be trustworthy, it should perform at an acceptable level of accuracy and be robust enough to withstand changes in the dataset such that a small change will not radically alter the performance. For recruitment, it is paramount that users are confident that systems are robust in this way;
- Privacy: Aside from issues of data stewardship, algorithms can process data in ways that reveal the nature of the data they have been trained on or that they are utilizing when they provide an output, such as a decision or recommendation. Here, concerns are with data leakage and, more generally, whether data minimization principles are being respected. Privacy concerns are particularly relevant in the context of recruitment given that individual and sensitive data relating to information such as race and gender is being processed.

### 3.4. Levels of Access

The final dimension to audit is the level of access an auditor has to a system. This level of access can range from white-box to black-box, where the levels of access being granted can determine which end of this continuum an audit falls within. White-box systems disclose the full model details, and audits of such systems have full access to the system being evaluated, allowing the auditor to have access similar to what the developer has. This kind of audit is the most accurate and has the highest level of detail. On the other hand, ‘black-box’/‘process-access’ systems only enable indirect observation, meaning that audits cannot directly access the system itself, resulting in a less accurate and less detailed audit. It is recommended that a low-level access audit is suitable for low-risk systems, whereas a high-level audit is suitable for high-risk systems such as those used in recruitment (European Commission 2020; German Data Ethics Commission 2018).

Critically, the verticals and points of assessment do not stand alone, but instead comprise a dynamic and highly interrelated complex. For example, after assessing and assuring a system on each vertical and aspect, model improvements and strategies to mitigate potential harm found in the audit could be introduced. These mitigation strategies should take into account the system as a whole as, for example, mitigation of a privacy risk may result in compromising a system’s fairness or explainability or vice versa. This is known as trade-off analysis, a practice that seeks to balance risk mitigation with maintaining the performance of the model. Importantly, risk mitigation and trade-off will be highly dependent on the individual model, as it can be impacted by factors including use cases, domain, the regulatory environment, risk appetite, and sector best practice. The appropriateness of a particular level of access to systems being used in the context of recruitment will be discussed subsequently, but for the moment it suffices to say that systems that have high levels of automation are higher-risk given that recruitment systems inherently use sensitive data.

### 3.5. Assurance

During the development of AI-driven recruitment tools, it is anticipated that by the end of the process, a mechanism will be in place to assure the system. This may include, for example, meeting a general and/or sector-specific standard, certification by a third party (if not the auditor—internal vs. external audit) and even insurance. As the movement toward model assurance is still in the early phase, the sector standards are unclear. However, we anticipate that the assurance will center primarily on the robustness and fairness verticals—which naturally aligns with the issues of fairness associated with recruitment contexts.

As is seen from the above, the process of assuring that an algorithmic system is trustworthy (assured, legal, ethical, safe, etc.) is multifaceted. A major challenge in this process is tracking all of the relevant information and ensuring that documentation is trustworthy. Information regarding the system needs to be organized in a systematic, rational and safe fashion that is accessible and retrievable. The most desirable practice for this would be automatic documentation of the information relevant to assurance and audit to allow it to be coherently and confidently investigated.

## 4. Contextual Factors

In order for the framework we have outlined above to be utilized appropriately, context is critical. Below, we outline a number of contextual factors that we anticipate will be of importance.

### 4.1. Human Oversight

In the algorithmic assessment literature, much is made of ensuring that humans remain in control of a system. Various schemes that have been formulated relate to the degree to which a human is in control and in what manner the human is involved:

- Human-in-the-loop: Involves a human capable of intervention in every decision cycle;
- Human-on-the-loop: Involves a human who is capable of intervention during the design cycle or who is monitoring its operation;
- Human-in-command: Involves a human capable of overseeing the overall activity of the system, with the ability to decide when and how to intervene in any particular situation.

These levels of control are in contrast to systems that are fully autonomous, and the risk level is proportionate to how autonomous the system is. In other words, the more human control, the smaller the risk. This is capitalized on by another approach that has suggested there is a need to have multiple people review a system before it is deployed (European Commission 2021).

### 4.2. Where in the Pipeline?

Recruitment services can be used in the context of a pipeline; in a large organization, a system could be used to filter a large number of applicants by removing applicants who fail to satisfy a particular condition or who are underqualified, while simultaneously allowing those who meet the relevant criteria to progress to the next stage of the recruitment process. In this context, given that the system is filtering at an early stage and with a relatively simple method, the risk is likely to be low. Contrastingly, if a system is being used at a later stage of an assessment process, such as to decide which candidates will attend a final interview, the audit will likely require greater inspection as there will be greater risk, both in terms of accuracy (robustness) and fairness.

### 4.3. What Is Explained and to Whom?

The right to an explanation on how a decision is made is provisioned in legislation such as GDPR; however, typical examples of this are credit scoring or policing applications. Recruitment is somewhat ambiguous with respect to a right to explanation. Here, the nature of the job the applicant is applying for and the stage of the recruitment process that they reach are likely to be important. For example, a candidate applying for a position in the civil service is more likely to be deemed to reasonably require an explanation if they are rejected, while an explanation is unlikely to be as important for those who are rejected from a graduate role that received thousands of applications. Thus far, we have spoken of explanations about decisions as a right; however, more generally, businesses may feel it is good practice to offer feedback to candidates by way of explanation of the factors influencing how positively and/or negatively their application was perceived. Moreover, this feedback may be given directly by recruitment services, which may be particularly useful for candidates who consistently receive unfavorable decisions, as explanations can relate to their general approach to applications instead of being relevant to a single, specific application. As such, explanation and transparency are highly context-dependent.

### 4.4. What Is Being Assessed?

A critical point of contention is the nature of the assessment with which the algorithmic system analyzes the candidate. Here, there are numerous forms of ‘data’ that can be used, including text in the form of written answers or resumes, as well as video assessments that extract data from facial expressions to perform sentiment analysis. The nature of what is assessed determines the risk level, where techniques that are considered more contentious, such as sentiment analysis of facial expressions, will be deemed to require greater mitigation strategies than low-risk strategies.

## 5. Conclusions

In this article, we utilized a systematic algorithm audit framework for recruitment systems (Koshiyama et al. 2021) and applied this in the context of recruitment. Following an introduction of some of the AI-based tools currently being used in recruitment and the advantages they can bring to candidates and hiring managers, we outlined the need for governance of the algorithms used by these technologies to ensure that they are being used in a fair and responsible way that does not disadvantage already vulnerable groups. Though our concern was mainly on an engineering-focused technical audit, we have discussed auditing in terms of levels of access to the algorithm and decision rules and specific assessment verticals such as data privacy, algorithm explainability and robustness, and ways to determine fairness or bias in a model. We also examined the level of human oversight involved in the development and application of these models, when audits would be most appropriate, and whether the decisions made by algorithms should be explained to candidates. Through this discussion, it should have become clear that such AI audits can render explicit the implicit/tacit knowledge in recruitment processes, which is critical for ensuring that a system is fair and accurate.

## Notes

1 Note that we use the term ‘algorithms’ to encompass a range of systems. At its most basic, an algorithm is simply a set of instructions designed to perform a specific task. Our main referent in this article is to data science algorithms, which include those that fall under AI paradigms such as machine learning or knowledge-based systems, used in human resources applications such as psychometric testing and recruitment (Koshiyama et al. 2020).

2 AI is also used to automate other human resources (HR) processes where decisions are being made about people (ex. posting job listings, matching to job roles, performance measurement, etc.). We will not explore these dimensions in this paper, but will rather tangentially touch upon them at points.
